# Cellular processing of gold nanoparticles: CE-ICP-MS evidence for the speciation changes in human cytosol

**DOI:** 10.1007/s00216-017-0749-0

**Published:** 2017-11-15

**Authors:** Joanna Legat, Magdalena Matczuk, Andrei R. Timerbaev, Maciej Jarosz

**Affiliations:** 1 Chair of Analytical Chemistry, Faculty of Chemistry, Warsaw University of Technology, Noakowskiego St. 3, 00-664 Warsaw, Poland; 2 Vernadsky Institute of Geochemistry and Analytical Chemistry, Kosygin St. 19, Moscow, 119991 Russia

**Keywords:** Capillary electrophoresis, Mass spectrometry, Gold nanoparticles, Human cytosol

## Abstract

The cellular uptake of gold nanoparticles (AuNPs) may (or may not) affect their speciation, but information on the chemical forms in which the particles exist in the cell remains obscure. An analytical method based on the use of capillary electrophoresis hyphenated with inductively coupled plasma mass spectrometry (ICP-MS) has been proposed to shed light on the intracellular processing of AuNPs. It was observed that when being introduced into normal cytosol, the conjugates of 10–50 nm AuNPs with albumin evolved in human serum stayed intact. On the contrary, under simulated cancer cytosol conditions, the nanoconjugates underwent decomposition, the rate of which and the resulting metal speciation patterns were strongly influenced by particle size. The new peaks that appeared in ICP-MS electropherograms could be ascribed to nanosized species, as upon ultracentrifugation, they quantitatively precipitated whereas the supernatant showed only trace Au signals. Our present study is the first step to unravel a mystery of the cellular chemistry for metal-based nanomedicines.

**Electronic supplementary material:**

The online version of this article (10.1007/s00216-017-0749-0) contains supplementary material, which is available to authorized users.

## Introduction

Gold nanoparticles (AuNPs) are widely researched as multifunctional theranostic agents, having, among other possible biomedical functions, the capability to exert anticancer efficacy by interacting with and altering tumor cells [1–4]. However, in contrast to metal-based drugs, for which the mechanisms of action have been at least in part elucidated, knowledge of the events involved in the cellular processing of AuNPs is limited. On the other hand, there is a great deal of experimental evidence that various nanoparticles are first adsorbed to the cell surface, then internalized and translocated to different cell compartments, and finally interacted with relevant subcellular structures to affect their function (see an excellent review by Mu et al. for a summary [5]). While still requiring comprehensive description and understanding, the scenario of the cellular uptake and targeting for AuNPs has much in common [6–9]. Much less is known, however, about the molecular mechanisms and chemical basis of AuNP–cell system interactions and, in particular, about pertinent nanosized metallic species participating in these interactions.

Given the intravenous route of administration, the foremost changes in the chemical state of AuNPs are due to interaction with proteins in the circulatory system [10–12]. Recently, it has been confirmed that for AuNPs engineered with different shapes, sizes, and surface modifications, the formation of the protein corona in human serum and, hence, the biological identity and response strongly depend on each of these constructive attributes [13–18]. The analytical methodology used in our studies [14–16] is based on coupling capillary electrophoresis (CE) to inductively coupled plasma mass spectrometry (ICP-MS) to selectively separate and sensitively detect the free and protein-bound AuNPs (see a recent review paper [19] proving the importance of using this combined technique in the field) and thus to get an idea of their speciation. It is safe to assume that the AuNPs enter the cell being covered with plasma proteins and then possibly underwent further compositional alterations, in the first instance, by interacting with the cytosolic components.

The present work focuses on unraveling these alterations using the same but slightly modified CE-ICP-MS setup (mainly, regarding the optimization of a capillary electrolyte composition). To simplify this challenging task, a single type of commercially available AuNPs with well-characterized functionalization and variable size was employed. Also, as a biosystem simplification, the nanomaterial, after being transformed into the protein-conjugated form in real human serum, was subjected to the action of cytosol fortified with various reducing and complexing agents (at their cancer cytosol concentrations) to mimic a cancer cytosol environment. This approach allowed us to ascertain the degradation of serum-induced corona on AuNPs followed by their conversion into a range of nanoforms with cytosolic molecules. This proof-of-concept finding will receive further investigation by using common proteomics methodology to decipher the cytosolic gold species.

## Materials and methods

### Chemicals and nanoparticle suspensions

All chemicals, including biological material (human serum from human male AB plasma and cytosol from human liver, pooled), were products of Sigma-Aldrich (St. Louis, MO, USA). Gold nanoparticle suspensions (5, 10, 20, and 50 nm in nominal diameters and 5.5, 9.4, 19.8, and 47.3 nm in mean diameters, respectively) were purchased from British Biocell International (Cardiff, UK) and stored in darkness at 4 °C. The Au concentration in aqueous suspensions was characterized by the producer as 63.2, 57.6, 56.6, and 56.8 mg L^−1^, respectively. High-purity water used throughout was obtained from an Elix Water Purification system (Millipore, Molsheim, France).

### Instrumentation

Analyses were carried out on a 7100 CE system (Agilent Technologies, Waldbronn, Germany) coupled to a 7500a ICP mass spectrometer (Agilent Technologies, Tokyo, Japan). Polyimide-coated fused silica capillaries (i.d. 75 μm; o.d. 375 μm; length 70 cm) were purchased from CM Scientific Ltd. (Silsden, UK). The liquid-introduction interface was based on a model CEI-100 nebulizer (CETAC, Omaha, NE, USA) equipped with a low-volume spray chamber and a crosspiece to merge the sheath liquid flow. Electrical circuit of the CE was completed via a grounded platinum wire. The capillary cassette and sample tray were thermostatted at 37 °C. The nebulizer performed in the self-aspiration mode using the sheath liquid to provide closing the electrical connection and to produce a fine aerosol. All signal quantifications were done in the peak area mode by monitoring the total ion current of ^197^Au during each CE run. The stability of CE-ICP-MS performance was controlled by measuring the normalized ^72^Ge signal during the postrun conditioning of the CE capillary as well as throughout the analysis. Analysis was only initiated when the signal was sufficiently high (cps > 4000) and stable (RSD < 2%). Instrumental control and data analysis were performed using Agilent ChemStation software. Operation conditions of the optimized CE-ICP-MS setup are summarized in Table 1.

**Table 1:** Optimal CE-ICP-MS operating parameters

CE system	
Capillary	Fused silica, i.d. 75 μm, o.d. 375 μm, length 70 cm
Capillary electrolyte	HEPES 40 mM, pH 7.4
Voltage	+ 15 kV
Temperature	37 °C
Sample injection	Hydrodynamic, 20 mbar, 5 s
Interface	
Sheath liquid	10 times diluted capillary electrolyte containing 20 μg L^−1^ Ge
Sheath liquid flow rate	10 μL min^−1^
ICP-MS system	
RF power	1380 W
Sample depth	7.0 mm
Plasma gas	15.0 L min^−1^
Nebulizer gas flow	1.2 L min^−1^
Monitored isotopes	^197^Au, ^72^Ge

Procedures for capillary initialization, conditioning each day and between runs, were the same as described before (see ref. 14). For the method development, samples were introduced by applying 20–50 mbar pressure for a specified time. Separations were performed by applying the voltage in the range 10–30 kV.

Samples were incubated before analysis in the CE autosampler at 37 °C, as regulated by a thermostat (Julabo, Seelbach, Germany). Ultrafiltration and ultracentrifigation were performed using an MPW-350R centrifuge (JW Electronic, Warsaw, Poland) and 100-kDa cut-off filters (in the case of ultrafiltration) obtained from Amicon Ultracel, Merck Millipore, Molsheim, France.

### Sample preparation

The concentration of AuNPs in all samples was adjusted to a dose at which the therapeutic effect was observed during radiotherapy of mice tumors [20], i.e., 1.35 g kg^−1^ Au, recalculated using the average human mass (70 kg) and blood volume (5 L). An aliquot of the stock solution of AuNPs was mixed with human serum diluted 1000 times with 10 mM phosphate buffer, pH 7.4, containing 100 mM NaCl (final Au concentration 19 mg L^−1^). To follow in vivo pharmacokinetic tests [21], the mixture was incubated at 37 °C for 45 min because this duration guarantees ca. 80% of the injected AuNPs to be eliminated from the bloodstream and transferred into targets. Likewise, this period allows the protein corona to form (although the protein-binding equilibrium is not necessarily attained [14]). The gold–protein conjugates, as well as unbound nanoparticles (where relevant), were separated from the unattached proteins and other matrix components (with molecular weight lower than 100 kDa) by ultrafiltration (10,000 rpm, 30 min). The high molecular weight fraction thus isolated was diluted to 500 μL by each of the four cytosol-like diluents shown in Table 2 (optimization of dilution factors for human cytosol is given in the Electronic supplementary material (ESM)), and the resultant solution was subjected to reverse ultrafiltration in order to being quantitatively removed from the ultrafiltration unit and then incubated at 37 °C. Aliquots were continuously taken for CE-ICP-MS analysis over 24 h. In parallel, the samples in preparation of which the step of mixing AuNPs with serum is omitted were incubated and analyzed. None of the preparations showed visible signs of particle aggregation. In examinations of the size distribution of cytosolic Au species, mixtures of AuNP conjugates with serum proteins and the diluted cancer cytosol (see Table 2, type B) were incubated for 2 h and centrifuged for 10 min at 3000 rpm, and both the supernatant and the precipitate (diluted with 20 or 480 μL 10 mM phosphate buffer pH 6.0, respectively) were analyzed by CE-ICP-MS.

**Table 2:** Biologically relevant media for treatment of the protein-bound AuNPs

Type	Medium	Simulating function
A	10 mM phosphate buffer (pH 6.0) containing 4 mM NaCl, 0.1 mM glutathione, 0.1 mM ascorbic acid, and 1 mM citric acid	Cancer cytosol-like solution (100 times diluted)
B	Standard of human cytosol diluted 100 times with 10 mM phosphate buffer (pH 6.0) containing 4 mM NaCl, 0.1 mM glutathione, 0.1 mM ascorbic acid, and 1 mM citric acid	Diluted cancer cytosol
C	Standard of human cytosol diluted 100 times with 10 mM phosphate buffer (pH 6.0), 4 mM NaCl	pH characteristic for diluted cancer cytosol
D	Standard of human cytosol diluted 100 times with 10 mM phosphate buffer (pH 7.4), 4 mM NaCl	Diluted normal cytosol

## Results and discussion

### Selection of CE electrolyte conditions

The objective of these trials was to find the background electrolyte suitable for the CE analysis of nanomaterials under simulated cancer cytosol conditions. It should be explained here that the intracellular environment of cancer cells is characterized by lower oxygen content and higher acidity compared to that of normal tissues. This is the consequence of an insufficient formation of new blood vessels in rapidly growing tumors, leading to poor blood and nutrient supply [22, 23]. As a result, cancer cells energetically depend rather on glycolysis and hence produce an excess of lactic acid, decreasing the pH from 7.4 to about 6.0, and a number of reducing and complexing agents such as glutathione, ascorbic acid, and citric acid [24–26]. A series of background electrolyte buffers with pH 6.0 was therefore examined (2-(*N*-morpholino)ethanesulfonic acid, piperazine-*N,N*′-bis(2-ethanesulfonic acid), phosphate, ammonium bicarbonate, ammonium carbonate, all tested in the range of 10–60 mM) for CE of 20 nm AuNPs. However, in all cases, irrespective of the magnitude of applied voltage (10–30 kV), a poor recovery of AuNPs (2–30%) or no gold-specific signals at all (even after 60 min of analysis) were observed. The most plausible explanation is behind the adsorption of AuNPs onto the capillary wall which also causes a change in the electroosmotic flow [27]. In this context, using alkaline electrolytes may be helpful as adsorption of the separands of interest would be reduced at higher pH values due to increased electrostatic repulsion [28]. For this reason, we decided to employ in the following experiments the HEPES buffer (4-(2-hydroxyethyl)piperazine-1-ethanesulfonic acid) of pH 7.4 that was successfully utilized for monitoring the speciation changes of AuNPs in human serum [14]. The separation parameters are presented in Table 1.

Under the optimized CE and ICP-MS conditions, the method’s analytical performance was assessed. A linear response between the peak area and gold concentration (for gold standard solution and suspensions of AuNPs of different sizes) was observed in the range 1.7–57.0 mg L^−1^ Au, with correlation coefficients higher than 0.99 (Fig. S1; ESM). The detection limit of Au, calculated for a signal/noise ratio of 3 using gold standard solution, was 1.0 μg L^−1^. The same sensitivity parameter for nanoparticle suspensions ranged from 1.4 (5 nm) to 4.2 μg L^−1^ (50 nm AuNPs).

### Speciation changes under cytosolic conditions

**Fig. 1:**
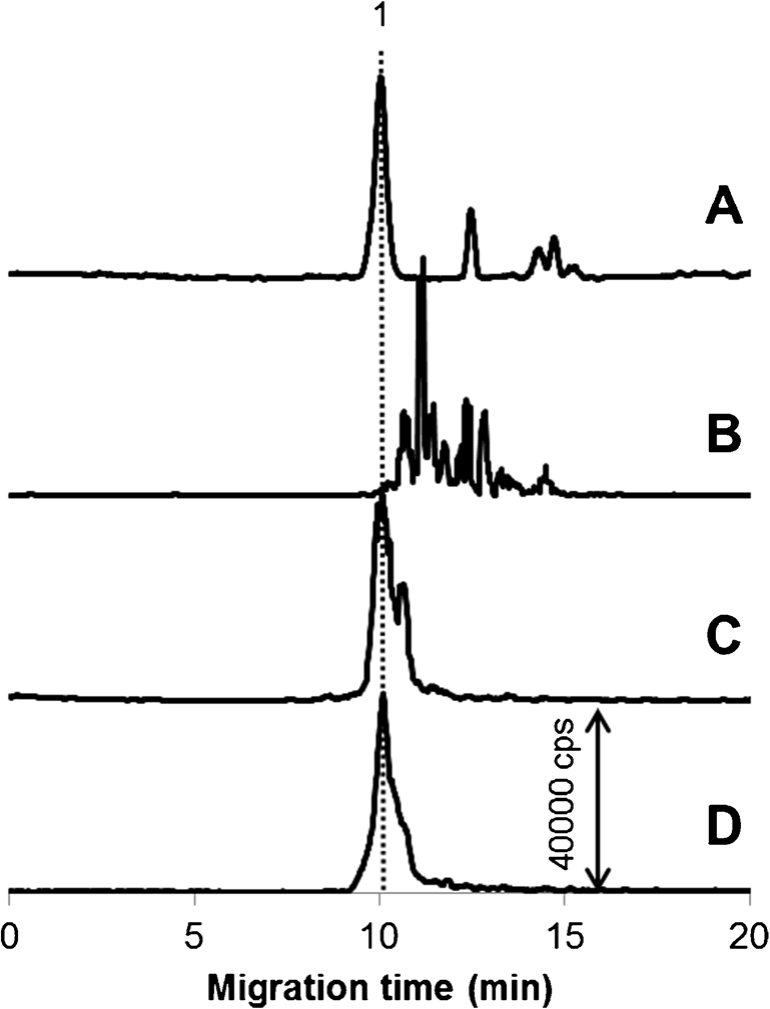
Electropherograms illustrating changes in the composition of the albumin conjugate of AuNPs (20 nm) under various conditions shown in Table 2 (traces A–D correspond to media A–D) after 30 min of sample incubation. Gold concentration, 19 mg L^−1^. The dotted line shows the migration time of the conjugate. Migration time repeatability for albumin conjugate chosen as a representative for this performance testing (peak 1) in samples A, C, and D is 6.9, 7.0, and 6.1% (RSD, *n* = 3), respectively. For CE-ICP-MS conditions, see Table 1

To gain insight into possible alterations of the AuNP–serum protein conjugates and the nature of newly formed species, the four types of samples, prepared using solutions detailed in Table 2, were examined following their incubation at physiological temperature for 30 min, and the results are summarized in Fig. 1. Note that the peak recorded at about 10 min (peak 1) belongs to the albumin conjugate, whereas the transferrin conjugates produced no signal. Apparently, in normal cytosol medium, the conjugate with albumin is not subject to compositional changes (at least those that would affect its electrophoretic behavior). Adjustment of the pH to that of cancer cytosol (pH 6.0) led to no major speciation changes, although the second comigrating peak is clearly visible. In contrast, the introduction of glutathione, ascorbic acid, and citric acid at their cancer cytosolic concentrations has an immense effect, causing a fast breakdown of the albumin conjugate and the formation of an array of novel Au species (Fig. 1, trace B). An upper electropherogram in Fig. 1 was recorded with the objective to exclude misinterpretation of these signals as belonging to gold aggregates and to prove their bioligand (and probably proteinaceous) nature. It is evident that in the sample made up without human cytosol, the AuNP–albumin conjugate is only partly decomposed (at ca. 15%) and there are only three newly formed peaks, two of which comigrate with the numerous peaks shown in trace B. This observation suggests that while somewhat disturbing the conjugate, the reducing/complexing agents prevent the nanoparticles from aggregation. Another important conclusion from the comparison of traces A and B is that the cytosolic proteins may play a key role in cellular processing (unless the contribution of other bioligands is overwhelming).

Adding to the interpretation of observed cytosolic transformations as not related to aggregation (agglomeration) processes is the fact that in none of the media tested (see Fig. S2; ESM) the appearance of aggregation-like signals for bare AuNPs takes place.

### Effect of the size of AuNPs on their speciation in simulated cancer cytosol

**Fig. 2:**
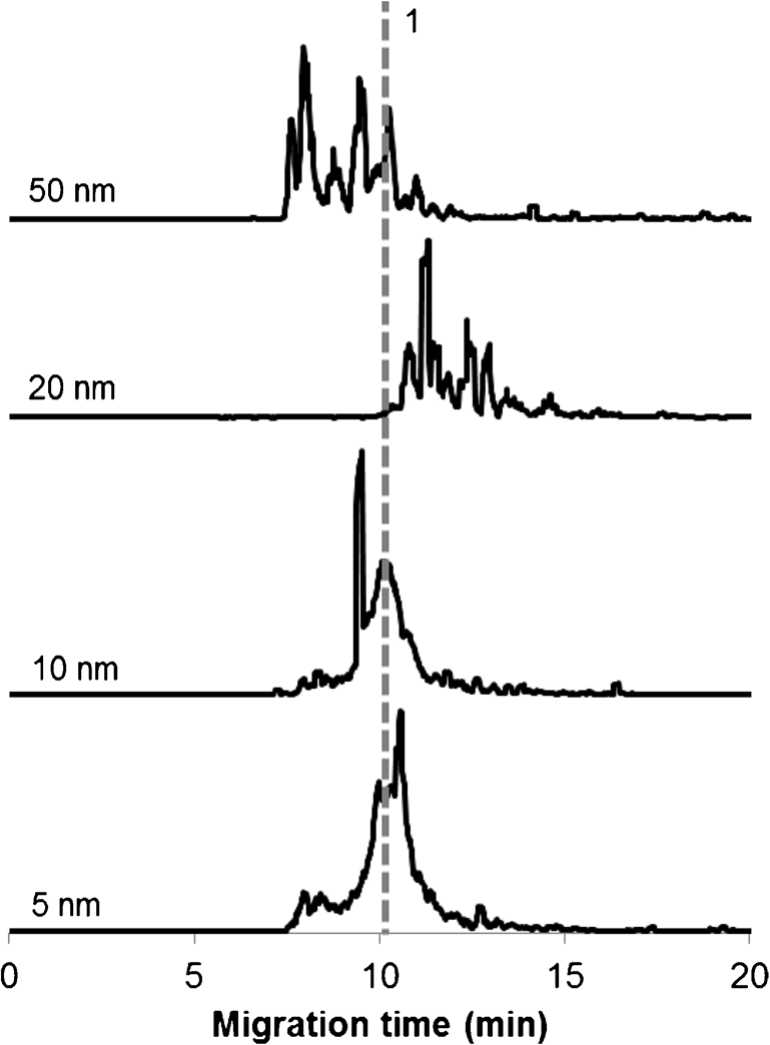
Effect of the particle size on compositional changes of serum–protein conjugates in simulated cancer cytosol after 20 min of sample incubation. Conditions, as in Fig. 1. Peak 1 belongs to the albumin conjugate. Repeatability of migration times for albumin conjugates is 6.8% (50 nm), 6.5% (10 nm), and 6.1% (5 nm) (RSD, *n* = 3)

Previous experiments were performed with AuNPs of one, intermediate size, i.e., 20 nm. However, it is known that the size of AuNPs has a strong influence on their speciation in human serum [14]. Therefore, it was deemed important to explore differently sized AuNPs exposed to simulated cancer cytosol (type B in Table 1). As can be seen in Fig. 2, the size of nanoparticles covered with serum proteins exhibits a significant impact on their cytosolic speciation, both in terms of the extent of losing the albumin corona (peak 1) and the number and electrophoretic behavior of the resulting species. The disintegration degree was calculated as a percent ratio of the summarized areas of all peaks other than the peak corresponding to the albumin conjugate to the peak area of all the signals presented in the electropherograms. It should be mentioned that neither the maximum degree of decomposition as shown in Table 3 nor the time required to reach it displays a correlation with the particle size. The only size-dependent relation observed was a higher resistance to disintegration of smaller particle conjugates. Indeed, the albumin conjugates of 10- and 5-nm AuNPs remain partly intact even after 24 h, whereas the conjugates of bigger nanoparticles (20 and 50 nm in diameter) disintegrate almost completely in less than 1 h after incubation in cancer cytosol. The binding profiles for these particles have much in common so that one can assume the same set of bioligands participating in their cytosolic transformation. The kinetic dependences recorded for all sizes of AuNPs and over the entire time span (up to 24 h) are presented in ESM, Fig. S3. Moreover, in ESM Fig. S4, the electropherograms are presented illustrating the speciation changes of 5- and 10-nm size AuNPs at the time of the experiments (on green are marked the electropherograms reflecting the reproducibility of results and registered for analogical samples but prepared in another day).

**Table 3:** Disintegration of the serum conjugates in simulated cancer cytosol

Particle size (nm)	Maximum percent of disintegration (*n* = 3)
5	91.2 ± 2.6
10	80.6 ± 3.1
20	99.5 ± 0.5
50	98.9 ± 1.1

### Characterization of newly formed Au species

**Fig. 3:**
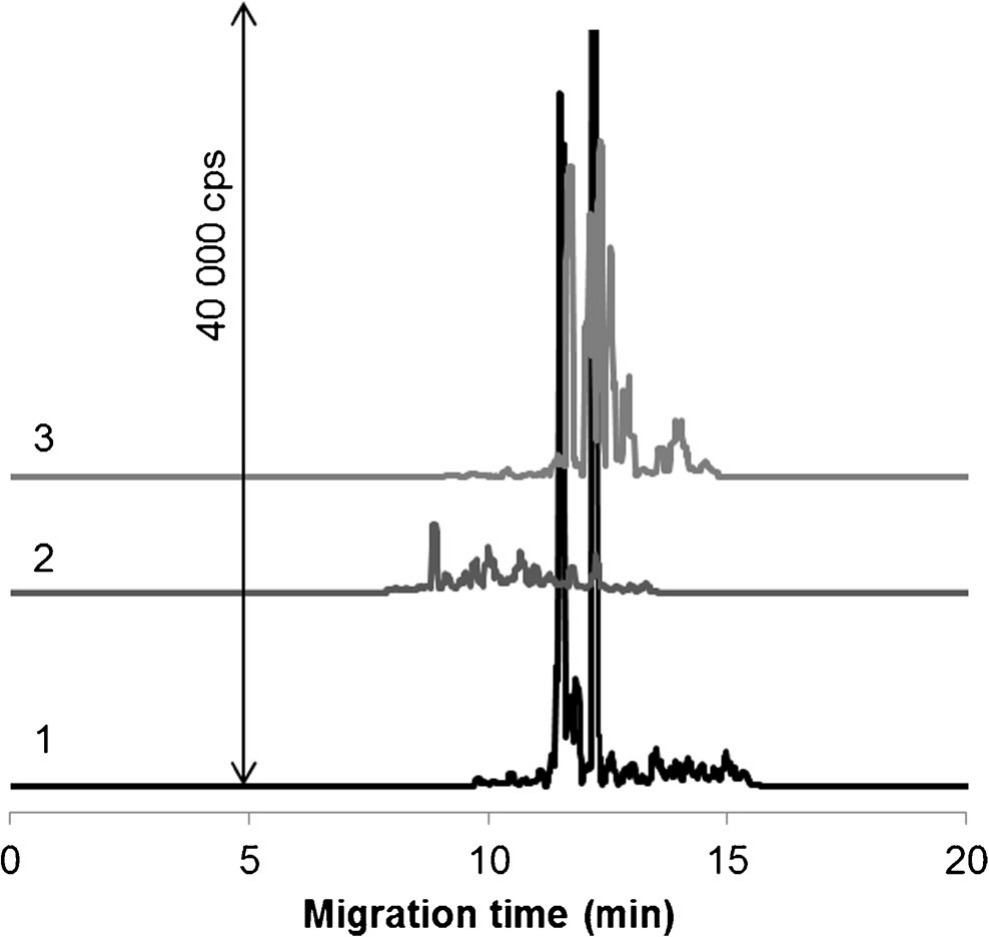
CE-ICP-MS analysis of cytosolic gold species (1) without sample centrifugation, (2) in supernatant, and (3) in precipitate after 2 h of sample incubation. AuNPs, 20 nm. Sample preparation and CE-ICP-MS conditions, see “Materials and methods”

The last question left to be answered in this study is whether all gold species are in the nanosized particulate form in simulated cancer cytosol or some of them are converted into an ionic form. Unfortunately, ultrafiltration applied to assess the size distribution of cytosolic species shown in Fig. 2 turned out to be ineffective because of their poor recovery from the membrane material. Ultracentrifugation was found to be more practicable and helped us to reveal that a great majority of Au species retain its nanostructure and become transferred into various conjugated forms with cytosolic molecules. As a proof-of-principle, Fig. 3 represents a comparison of electropherograms of the separated fractions acquired for 20 nm AuNPs. At this stage, it seems too early to speak of the identity of cytosolic nanospecies, although generally the high affinity of AuNPs toward proteins and an extreme number of proteins, occurring in the cytosol, raises the possibility of their proteinaceous nature. Also, the data of Arvizo et al., proving that in ovarian cell lysates the surface of AuNPs is covered with proteins, should not be overlooked [29].

## Conclusions

Our work proved that similar to metal-based drugs, the speciation changes of AuNPs (as conjugates with serum proteins at extracellular circumstances) would take place upon arrival to cancer cell. Compositional alterations of AuNPs in the presence of cytosolic constituents were found to depend on the particle size and the time for which the AuNP–serum conjugates are exposed to simulated cancer cytosol. However, this important biomedical implication requires further investigation as only simulated medium was considered here and no exact characterization of the gold species was obtained (other than these are multiple particulate forms conjugated with cytosolic molecules). Therefore, a future methodological improvement will be to combine CE with electrospray ionization-MS/MS. This combination is expected to be able to identify the Au species in real cancer cytosol, directly or after sample enzymatic digestion, and possibly to recognize the intracellular targets of AuNPs.

## Electronic supplementary material

ESM 1 (PDF 380 kb)
