# Supplementary material for: Cellular processing of gold nanoparticles: CE-ICP-MS evidence for the speciation changes in human cytosol
Source: Anal Bioanal Chem. 2017 Nov 15;410(3):1151–6. doi: 10.1007/s00216-017-0749-0 (PMC5775379; doi:10.1007/s00216-017-0749-0)
Supplement: Supplementary file 1 — (PDF 380 kb) [file 216_2017_749_MOESM1_ESM.pdf]

Analytical and Bioanalytical Chemistry

Electronic Supplementary Material

Cellular processing of gold nanoparticles: CE-ICP-MS evidence for the speciation changes in human cytosol

Joanna Legat, Magdalena Matczuk, Andrei R. Timerbaev, Maciej Jarosz

Optimization of cytosol dilution factor

The dilution factor for human cytosol was varied from 20 to 200 with due account for preventing the adsorption of cytosol components (e.g. proteins) and their conjugates with AuNPs onto the capillary wall. Used as a diluent was 10 mM phosphate buffer (pH 6.0), containing glutathione, ascorbic, and citric acid (type B in Table 2), whose concentrations – after dilution – correspond to 10, 10, and 100 mM, respectively, to simulate cancer cell cytosol, (i.e., about 100 times higher than in normal cytosol) [1,2]. After incubation for 30 min at physiological temperature, the samples were analyzed by CE-ICP-MS. The obtained recoveries are shown in Table S1. Not surprisingly, at lower, 20–50-fold dilutions, with higher concentrations of cellular proteins introduced into the separation capillary, the recoveries are unsatisfactory. The highest recovery (83.5%) was obtained for the cytosol diluted 100-times and this dilution factor was chosen for further investigations.

Table S1 Effect of the cytosol dilution factor on the metal recovery

Dilution factor	20	50	100	200
Recovery (%), $n = 3$	59.7 ± 3.4	65.1 ± 6.3	83.5 ± 5.3	80.3 ± 4.4

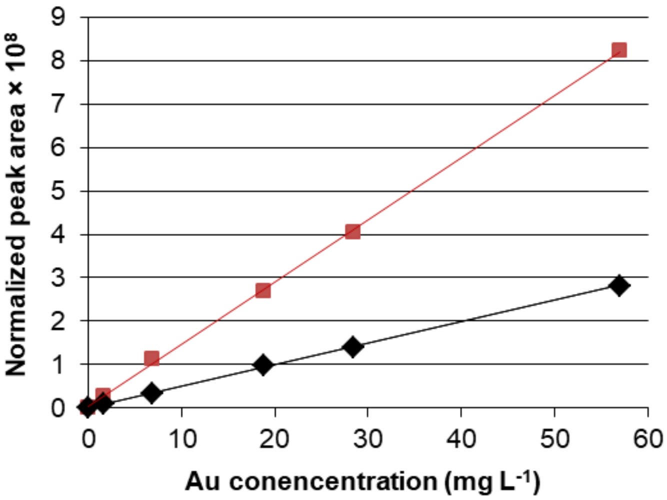

Fig. S1 Calibration curves for gold (red line) and 20 nm AuNPs (black line). The correlation equations and coefficients (in parentheses) are y = 14319200x + 2743470 (0.999) and y = 4935060x + 1028200 (0.999), respectively

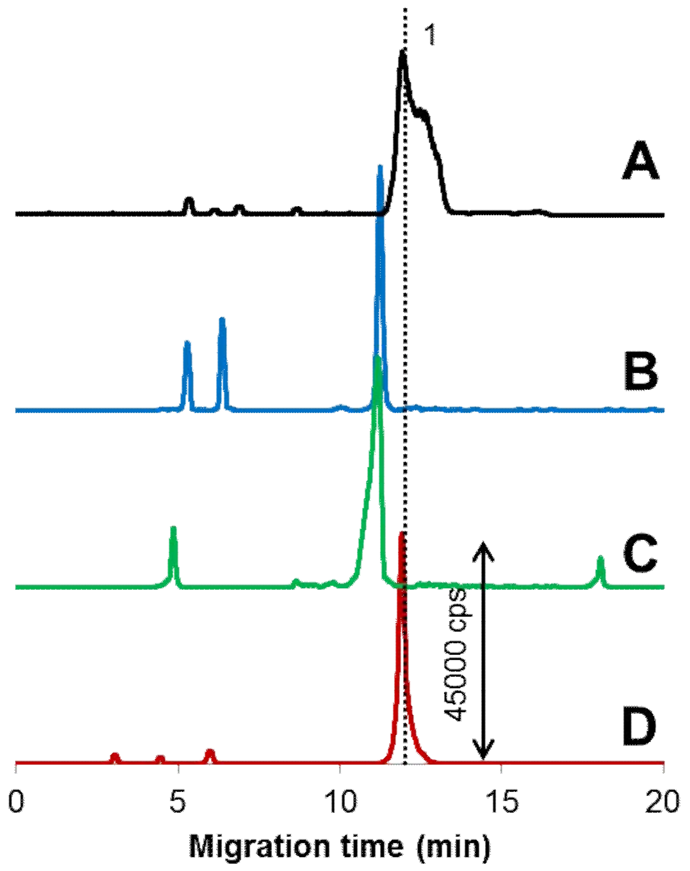

Fig. S2 Electropherograms illustrating changes in the composition of bare AuNPs (20 nm) under various conditions shown in Table 2 (traces A–D correspond to media A–D). Gold concentration, 19 mg L^{-1}. Peak assignment: 1 – AuNPs. For CE-ICP-MS conditions, see Table 1

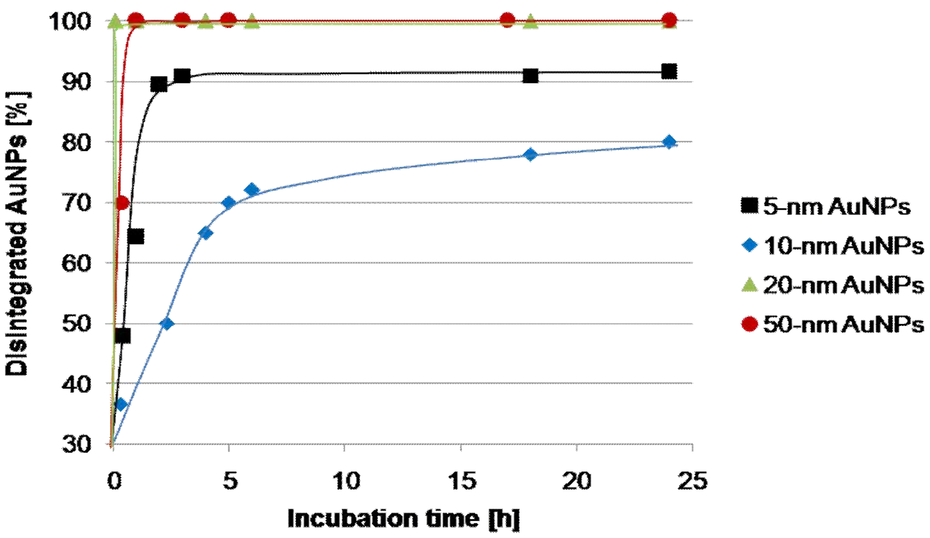

Fig. S3 Kinetics of decomposition of the serum proteins conjugates under simulated cancer cytosol conditions (Type B, see Table 2)

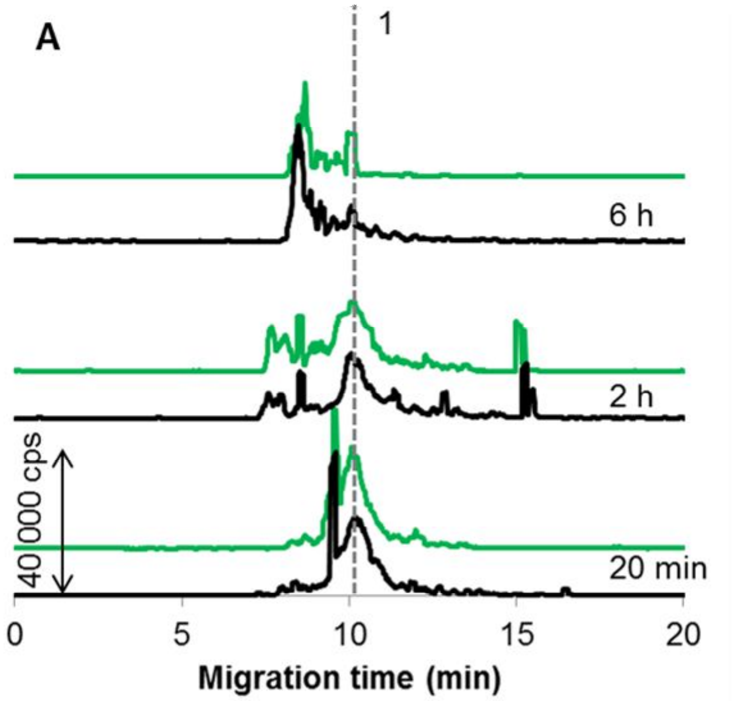

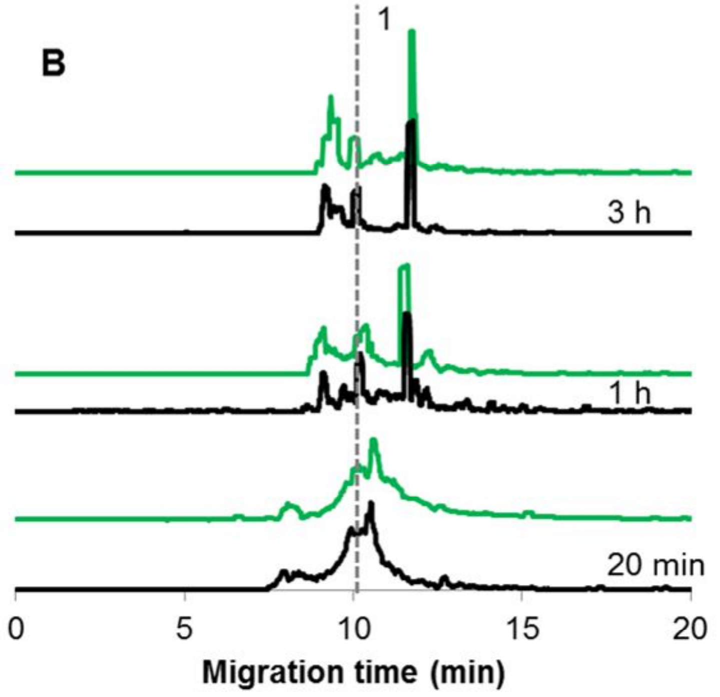

Fig. S4 Speciation changes of the conjugates of (A) 10 nm and (B) 5 nm AuNPs in simulated cancer cytosol (type B, see Table 2) after various incubation times. Gold concentration, 19 mg L^{-1}. Peak 1 belongs to the albumin conjugate. Repeatability of migration times is shown in Table S2. Long-term reproducibility of results is presented on green (analogical samples but prepared in another day)

Table S2 Repeatability of migration times for albumin conjugates in simulated cancer cytosol (n = 3)

Size (nm)	Incubation time	RSD (%)
5	20 min	6.5
	2 h	6.1
	6 h	6.0
10	20 min	6.1
	1 h	6.4
	3 h	5.5

References

1. Ortega AL, Mena S, Estrela JM. Glutathione in cancer cell death. Cancers. 2011;3:1285–1310.
2. Pastore A, Federici G, Bertini E, Piemonte F. Analysis of glutathione: implication in redox and detoxification. Clin Chim Acta. 2003;333:19–39.
